# Effect of Metformin Use on Survival in Resectable Pancreatic Cancer: A Single-Institution Experience and Review of the Literature

**DOI:** 10.1371/journal.pone.0151632

**Published:** 2016-03-11

**Authors:** Chenwi M. Ambe, Amit Mahipal, Jimmy Fulp, Lu Chen, Mokenge P. Malafa

**Affiliations:** 1 Department of Gastrointestinal Oncology, H. Lee Moffitt Cancer Center and Research Institute, Tampa, Florida, United States of America; 2 Department of Biostatistics, H. Lee Moffitt Cancer Center and Research Institute, Tampa, Florida, United States of America; University of Florida, UNITED STATES

## Abstract

Observational studies have demonstrated that metformin use in diabetic patients is associated with reduced cancer incidence and mortality. Here, we aimed to determine whether metformin use was associated with improved survival in patients with resected pancreatic cancer. All patients with diabetes who underwent resection for pancreatic adenocarcinoma between 12/1/1986 and 4/30/2013 at our institution were categorized by metformin use. Survival analysis was done using the Kaplan-Meier method, with log-rank test and Cox proportional hazards multivariable regression models. For analyses of our data and the only other published study, we used Meta-Analysis version 2.2. We identified 44 pancreatic cancer patients with diabetes who underwent resection of the primary tumor (19 with ongoing metformin use, 25 never used metformin). There were no significant differences in major clinical and demographic characteristics between metformin and non-metformin users. Metformin users had a better median survival than nonusers, but the difference was not statistically significant (35.3 versus 20.2 months; *P* = 0.3875). The estimated 2-, 3-, and 5-year survival rates for non-metformin users were 42%, 28%, and 14%, respectively. Metformin users fared better with corresponding rates of 68%, 34%, and 34%, respectively. In our literature review, which included 111 patients from the two studies (46 metformin users and 65 non-users), overall hazard ratio was 0.668 (95% CI 0.397–1.125), with *P* = 0.129. Metformin use was associated with improved survival outcomes in patients with resected pancreatic cancer, but the difference was not statistically significant. The potential benefit of metformin should be investigated in adequately powered prospective studies.

## Introduction

Pancreatic cancer is the 10th most common cancer in the United States, with an estimated incidence for 2014 of 46,420 [1]. Unfortunately, pancreatic cancer is associated with poor prognosis and is the fourth most common cause of cancer death in the United States, with an estimated 39,590 patients expected to die from the disease in 2014 [1]. Even in patients who present with early disease and undergo margin-negative resection, the 5-year survival rate is only 24%. For patients who present with unresectable disease, the 5-year survival rate is worse at 2% [2]. These dismal survival data highlight the need for better treatment strategies for the management of pancreatic cancer. The surgical treatment of pancreatic cancer has improved dramatically over the past 20 years; however, further refinements in surgical techniques are unlikely to result in major survival benefits.

Most patients who develop recurrent disease after an R1 resection have distant metastases as opposed to local recurrence. This is usually the result of resistance of cancer cells to chemotherapy. Advances in the treatment of resectable pancreatic cancer will most likely result from the development of drugs that can prevent relapse, whether it is local or distant. Another approach is to use novel medications that target different pathways such as metabolism, compared to traditional chemotherapy medications. These drugs may be able to eliminate chemotherapy-resistant cells and/or help to prevent relapse. One drug that seems to hold promise for the latter approach is metformin.

Metformin, an anti-diabetic drug, has been associated with chemoprevention, with a decreased incidence shown in multiple cancer types, including breast, prostate, pancreas, and hepatocellular carcinoma [3–8]. Metformin has also been shown to have chemotherapeutic potential, with patients using metformin who develop cancer having improved survival compared with non-users of metformin [3–6,8–13]. Specifically, in pancreatic cancer, the use of metformin has been associated with a decreased incidence of disease, suggesting a chemopreventive effect of metformin [12,14]. In addition, a recent single-institution study reported that metformin use was associated with better survival in patients diagnosed with pancreatic cancer [15]. Metformin is believed to exert its anti-neoplastic activity through activation of liver kinase B1, which leads to activation of AMP-activated protein kinase. AMP-activated protein kinase in turn controls the mammalian target of rapamycin (mTOR) growth regulatory pathway [16]. The synthesis of cell growth factors involved in the regulation of cell growth and angiogenesis is influenced by the mTOR pathway [17]. After a margin-negative surgical resection, metformin could possibly have a role in the prevention of growth of microscopic foci of metastases.

In this study, we evaluated the effect of metformin use on survival in patients with resectable pancreatic cancer. We focused on patients with resectable pancreatic cancer who have the best survival and will likely derive a higher benefit with metformin use as a chemopreventive agent. We also performed a literature review using data from our study and a previous study that looked at the same question given the relatively small sample size of each study.

## Patients and Methods

We queried a prospectively maintained database for all patients who had undergone pancreatic resection between 12/1/1986 and 4/30/2013, with a total of 939 patients found. Patients in this database had provided written informed consent to have their medical information included. From this list, we identified patients who had undergone resection for adenocarcinoma. Only patients with a preoperative diagnosis of diabetes mellitus and stage I and II disease who underwent surgical resection were included. A chart review was then conducted to collect information, including age, race, sex, date of diagnosis, date of surgery, tumor location, type of surgery, margin status, metformin use, body mass index (BMI), CA 19–9 level, number of lymph nodes resected and number positive, pathologic TNM classification, date of recurrence, date of death where applicable, and last date of follow-up. Death was confirmed by either the medical record or review of the social security death index. Patients who were alive were censored at the last follow-up date. Recurrence was determined either radiologically or based on pathologic review of biopsied tissue. Our study received approval from the University of South Florida Institutional Review Board.

An exhaustive search of PubMed was performed using the keywords pancreatic adenocarcinoma, metformin, and diabetes mellitus in various combinations. We restricted the search to studies with human subjects in the English language. The abstracts of the search results were reviewed. The references of relevant articles were reviewed for further articles. We were able to find only one study that reported a single-institution experience. This study evaluated the association between metformin use and survival in patients with all stages of pancreatic cancer. For the literature review, we included this second study, utilizing the data pertaining to patients with resectable disease only.

### Statistical analysis

We compared clinical and pathological factors to metformin treatment. We tested the relationship using chi-square test for categorical clinical and pathological factors and Wilcoxon rank sum test for continuous clinical and pathological factors, both with the exact method using Monte Carlo estimation. Kaplan-Meier curves were created for both overall survival and progression-free survival and log-rank tests were used to compare treatment for overall survival analysis. Multivariable overall survival models were fit using Cox proportional hazard models. We found our final models using backward selection, with a removal alpha of 0.05. All *P* values are two-sided, unless otherwise stated, and considered statistically significant at the 0.05 level. All statistical analyses were performed using SAS (version 9.3; SAS Institute; Cary, NC).

We obtained the hazard ratio (HR) and its variance for patients with resectable pancreatic cancer from the corresponding author of the previously published study. We also calculated the HR for our study. The HRs summarized the time to event (overall survival) for each study. Comprehensive Meta-Analysis Version 2.2 was used for the analyses. Due to the small number of studies we could consider, we were unable to address publication bias or heterogeneity.

## Results

Forty-four patients were identified who met all of the inclusion criteria. Fifty-seven percent (25/44) did not take metformin, while 43% (19/44) did. There were 14 female (31.8%) and 30 male patients (68.2%). The patients were predominantly white (93.2%). The mean age at diagnosis was 68 years, with a range of 40–88 years. The mean maximum tumor measurement was 3.5 cm (range, 1–9 cm). Weight information was unavailable for 5 patients. The mean BMI was 29.1 (range, 22.4–60.7), with 13/39 patients being overweight (BMI > 30). Mean CA 19–9 at diagnosis was 5,897.84, with a range of 1.5–172,000. The mean number of regional nodes examined was 15.16 (range, 2–49). The average number of positive nodes was 1.64 (range, 0–12). In addition, the mean albumin level was 3.94 (range: 2.2–5.1). Eighty-four percent (37/44) of the tumors were located in the head, 4/44 (9%) were located in the tail, and 3/44 (7%) were located in the body of the pancreas. Eighteen percent of patients (8/36) had stage I disease and 36/44 (81.8%) had stage II disease. Margin status was available for 42 patients, with 37 having negative margins (88.1%) and 5 having positive margins (11.9%). There were no significant differences between the two groups with regard to age, gender, race, BMI, tumor grade, margin status, stage, surgery type, number of lymph nodes harvested, number of positive lymph nodes, ECOG status, serum albumin, and diabetes duration. Further details of the patient clinical characteristics are shown in Tables 1 and 2. There were no perioperative deaths in either group. All patients went on to receive adjuvant chemotherapy and radiation, which is the standard of care at our institution.

**Table 1:** Clinicopathologic characteristics of the patients by group.

Variable	All Patients (N = 44)	Metformin (N = 19)	Non-Metformin (N = 25)	*P* Value
**Gender, N (%)**				1.0000
** Female**	14 (31.8)	6 (31.6)	8 (32)	
** Male**	30 (68.2)	13 (68.4)	17 (68)	
**Age at diagnosis, N (%)**				1.0000
** <60 years**	7 (15.9)	3 (15.8)	4 (16)	
** 60+ years**	37 (84.1)	16 (84.2)	21 (84)	
**Race, N (%)**				
** Non White**	3 (6.8)	1(5.3)	2 (8)	
** White**	41 (93.2)	18 (94.7)	23 (92%)	
**BMI, N (%)**				0.4152
** ≤25**	10 (25.6)	5 (33.3)	5 (20.8)	
** 25–30**	16 (41%)	7 (46.7)	9 (37.5)	
** >30**	13 (33.3%)	3 (20)	10 (41.7)	
**Margin status, N (%)**				1.0000
** Negative**	37 (88.1)	15 (88.2)	22 (88%)	
** Positive**	5 (11.9)	2 (11.8)	3 (12%)	
**ECOG, N (%)**				0.2807
** 0**	34 (77.3)	13 (68.4)	21 (84)	
** 1**	10 (22.7)	6 (31.6)	4 (16)	
**Stage, N (%)**				
** I**	8 (18.2)	5 (26.3)	3 (12)	0.2688
** II**	36 (81.8)	14 (73.7)	22 (88)	
**Grade/differentiation, N (%)**				0.8450
** Poor**	12 (27.3)	5 (26.3)	7 (28)	
** Moderate**	19 (43.2)	7 (36.8)	12 (48)	
** Well**	8 (18.2)	4 (21.1)	4 (16)	
** Not reported**	4 (11.4)	3 (15.8)	2 (8)	
**CA 19–9, N (%)**				0.2370
** 0–35**	8 (23.5)	1 (7.7)	7 (33.3)	
** >35–1000**	15 (44.1)	7 (53.8)	8 (38.1)	
** >1000**	11 (32.4)	5 (38.5)	6 (28.6)	
**Albumin, N (%)**				0.0617
** ≤3.5**	6 (15)	0 (0)	6 (25)	
** >3.5**	34 (85)	16 (100)	18 (75)	
**Nodes examined, N (%)**				0.7631
** <11**	17 (38.6)	8 (42.1)	9 (36%)	
** >11**	27 (61.4%)	11 (57.9)	16 (64%)	
**Positive nodes, N (%)**				0.3653
** 0**	21 (47.7)	11 (57.9)	10 (40)	
** 1+**	23 (52.3)	8 (42.1)	15 (60)	
**Surgery, N (%)**				
** Whipple**	8 (18.2)	3 (15.8)	5 (20)	
** Non Whipple**	36 (81.8)	16 (84.2)	20 (80)	
**Diabetes duration, N (%)**				0.4676
** 0–2 years**	13(46.4)	4 (36.4)	9 (52.9)	
** >2 years**	15 (53.6)	7 (63.6)	8 (47.1)	

BMI, body mass index.

**Table 2:** Univariate analysis of survival.

Variable	Reference	Level	HR (95% CI)	Overall P Value
Age at Diagnosis	<60	60+	2.03 (0.59,6.96)	0.2588
BMI	≤25	25–30	0.34 (0.12,1)	0.1382
	≤25	>30	0.55 (0.2,1.53)	
BMI	≤25	>25	0.59 (0.23,1.53)	0.2821
Albumin	<3.5	3.5+	0.76 (0.25,2.26)	0.6190
Regional Nodes Examined	<11	11+	0.91 (0.4,2.07)	0.8244
Regional Nodes Positive	0 Nodes Positive	1+ Nodes Positive	1.96 (0.86,4.46)	0.1100
Metformin	No	Yes	0.7 (0.31,1.59)	0.3893
Gender	Female	Male	1.08 (0.45,2.6)	0.8630
Race	Not White	White	0.5 (0.15,1.72)	0.2713
Margin Status	Negative	Positive	1.31 (0.45,3.84)	0.6220
ECOG	0	1	1.15 (0.48,2.77)	0.7493
Stage	I	II	0.72 (0.27,1.92)	0.5067
Grade/Differentiation	Poorly Diff	Moderately Diff	0.47 (0.18,1.23)	0.2411
	Poorly Diff	Well Diff	0.94 (0.34,2.6)	
Grade/Differentiation (Grouped)	Poorly Diff	Moderately/Well Diff	0.61 (0.26,1.41)	0.2444
CA 19–9	0–35	>35–1000	1.55 (0.32,7.53)	0.8517
	0–35	>1000	1.55 (0.3,7.95)	
CA 19–9	0–35	>35	1.55 (0.34,7.09)	0.5710
Surgery Type	Not Whipple	Whipple	0.29 (0.11,0.76)	0.0120
Diabetes Duration	0–2 Years	>2 Years	2.06 (0.74,5.73)	0.1652

HR, hazard ratio.

**Fig 1:**
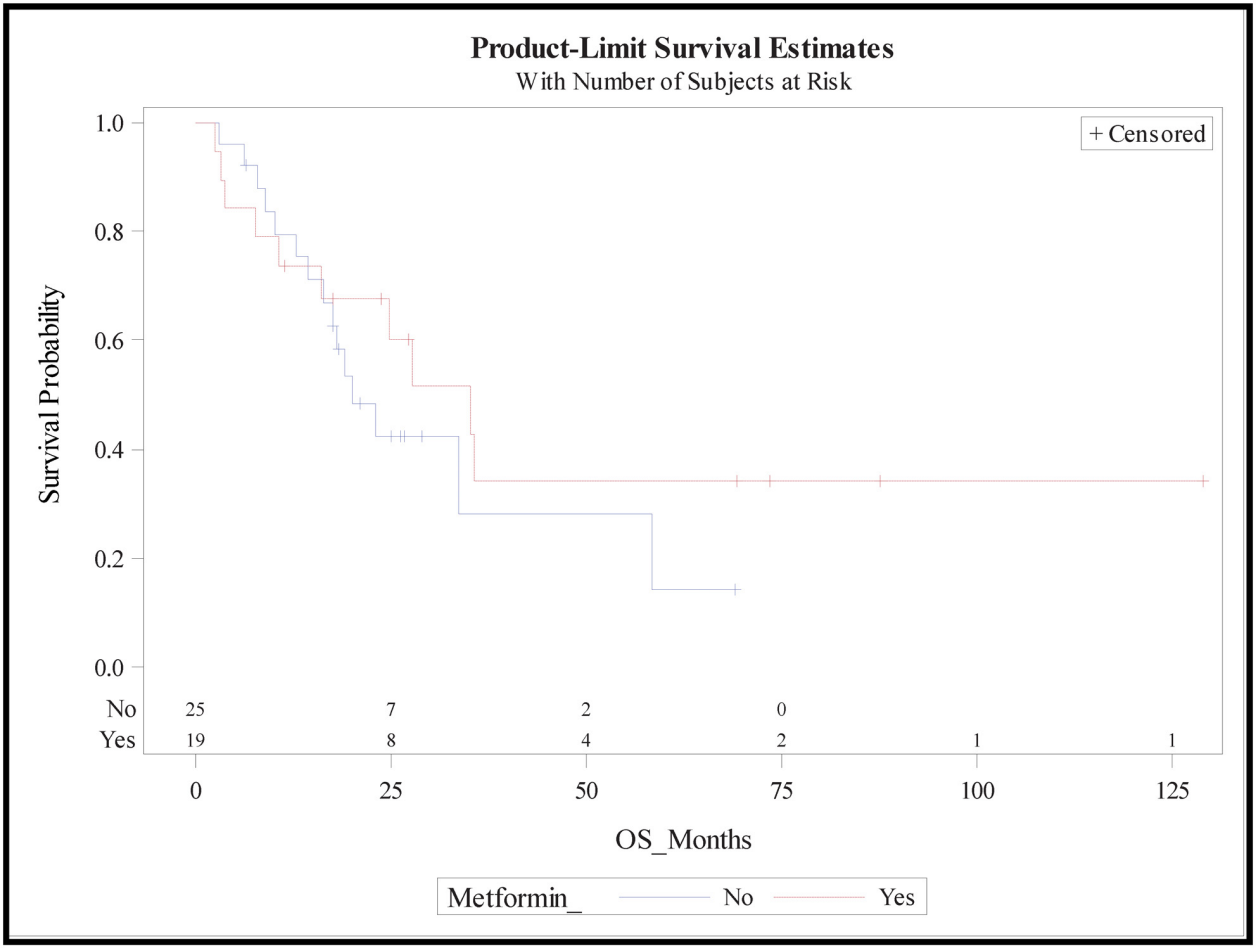
Kaplan-Meier survival curves for the metformin group (dashed line) and non-metformin group.

The median follow-up for the study was 19 months (range, 2–129). For the non-metformin group, median survival was 19.3 months (95% CI, 14.4–58.2), while for the metformin group it was longer at 29.8 months (95% CI 10.0, not estimable) (Fig 1). There were no significant differences in survival between the two groups with HR of 0.54 (95% CI, 0.16–1.86). The estimated 2-year survival was improved in the metformin group compared with the non-metformin group, at 0.61 (95% CI, 0.34–0.79) and 0.42 (95% CI, 0.21–0.62), respectively. This trend held at 3 years with estimated survival of 0.35 (95% CI, 0.12–0.59) and 0.28 (95% CI, 0.07–0.55) in the metformin and non-metformin groups, respectively. The five-year estimated overall survival was again better in metformin users at 34% (95% CI, 12–59%) compared with 14% (95% CI, 1–44%) in non-metformin users. On univariate analysis, none of the variables examined, including margin status, regional nodes examined, number of regional nodes positive, age at diagnosis, BMI, type of surgery, diabetes duration, CA 19–9 grade, and stage, was associated with improved survival. There were no differences in the results on multivariable analysis.

**Fig 2:**
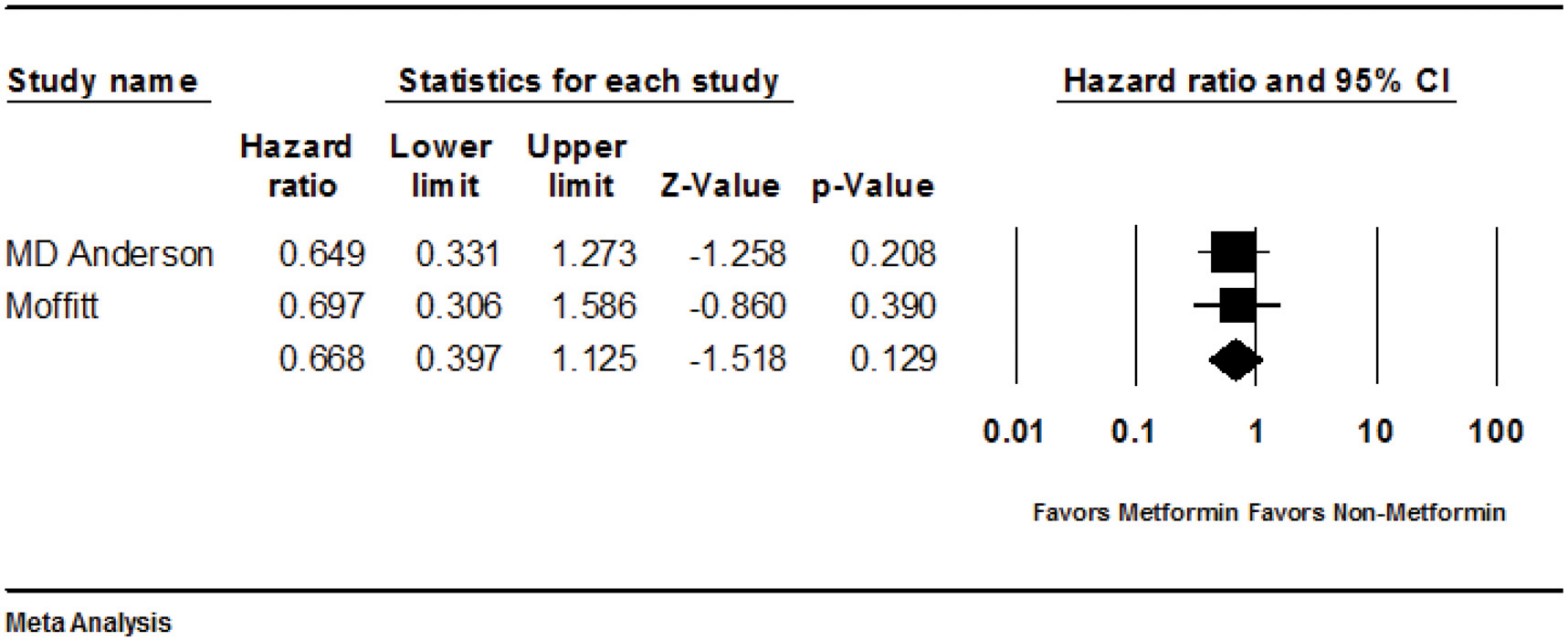
Forest plot of analysis.

The study by Sadeghi et al reported data on 67 patients with resectable pancreatic cancer and evaluated clinical outcome by metformin use. There were 27 patients in the metformin users group and 47 patients in the non-metformin users group. The HR of survival for the metformin group compared with the non-metformin group in resectable patients was 0.65 (95% CI, 0.33–1.27; *P* = 0.209). We performed an analysis of the two studies. The total sample size for this analysis was 111 patients, with 46 patients assigned to the metformin and 65 patients to the non-metformin group. In the analysis, the HR for the metformin group compared with the non-metformin group was 0.67 (95% CI 0.40–1.12; *P* = 0.129) (Fig 2).

## Discussion

The relationship between diabetes mellitus and pancreatic cancer is complex. Type 2 diabetes mellitus has been shown to be a risk factor for pancreatic cancer [7]. In addition, close to 8% of patients with pancreatic cancer are found to have new onset type 2 diabetes mellitus or impaired glucose tolerance at the time of diagnosis or shortly thereafter [18,19]. The pathophysiologic derangements that are responsible for the development of diabetes mellitus have also been associated with an increased risk for cancer development [20]. Studies using animal models have also shown that the increased levels of insulin that result from insulin resistance compounded by exogenous insulin administration lead to an increased level of vulnerability to chemically induced carcinogenesis [21,22]. Metformin may have improved outcome by improving insulin resistance, leading to lower levels of circulating insulin. In addition to decreasing circulating levels of insulin, metformin has been shown to have direct antitumor activities. Studies using cancer cell lines have shown that metformin impairs growth of cancer cells [23–27]. Some of these studies also examined human tumors xenografted onto nude mice, which showed that metformin retains its antitumor activity in vivo [26,28]. Both insulin and insulin-like growth factor 1 have proliferative and anti-apoptotic effects via the mitogen-activated protein kinase. Reduction of circulating levels of insulin is one postulated anti-tumor mechanism of action of metformin. Another anti-tumor mechanism of action of metformin is via mitogen-activated protein kinase activation in the presence of liver kinase B1 [27]. This leads to inhibition of mTOR, which is involved the control of synthesis of cell growth factors involved in the regulation of cell growth and angiogenesis and is influenced by the mTOR pathway [17,27]. In addition, metformin has been shown to exert its anti-neoplastic effect by enhancement of apoptosis induction [24] and finally cell cycle arrest [23]. More recently, Nair et al showed that treatment of a human pancreatic cancer cell line with metformin resulted in downregulation of the insulin-like growth factor 1 receptor, which in turn inhibited mTOR. The other effect was to decrease epidermal growth factor receptor, which resulted in inhibition of the Ras oncogene [29]. Furthermore, a recent study of diet-induced obese mouse demonstrated that addition of metformin not only normalized insulin levels but also enhanced anti-tumor immunity [30].

In our study, there was a non-significant trend toward improved survival with the use of metformin in patients with resectable pancreatic cancer who were diabetic. The median overall survival of 10.4 months was longer in those who took metformin than in those who did not. Furthermore, the long-term survival was higher in the metformin group than in the non-metformin group, with 5-year survival rates of 34% and 14%, respectively. These results are consistent with results from a previously reported study. In that study, the median survival in patients who received metformin was 31.0 months versus 21.4 months in patients who did not [15].

One of the limitations of our study includes its retrospective design. In addition, we did not evaluate patients with impaired glucose tolerance who may have received metformin. Lastly, the number of patients was small, limiting the power of our study. All patients received adjuvant chemotherapy and radiation. However, we did not examine the specific regimens and their distribution between the groups. Likewise, we did not look at the difference in use of other anti-diabetic medications including the use of insulin. On the other hand, our study had several strengths. First, all of the patients had surgery at a single high-volume center with experienced surgeons. Likewise, pathology review was at a single institution by the same group of pancreatic cancer pathologists, thus ensuring uniformity in the pathologic reports. Although the overall number of patients was low, it is very close to the number reported by the other study, which was done at a high-volume institution like ours. This suggests that it will take a multi-institutional study to accumulate a large volume of patients to achieve a significantly higher power. There were also no significant differences between the two groups, other than the use of metformin. The results of both studies were consistent, as was the literature analysis, which showed a 43% reduction in mortality in metformin users. This suggest that the association of improved survival with metformin use is likely real.

Two studies that used a metformin-containing regimen to treat pancreatic cancer were recently presented at the 2014 ASCO meeting [31]. The first was a phase II trial that looked at the use of a metformin-containing regimen (metformin and paclitaxel) as second-line therapy in advanced pancreatic cancer. The study did not meet the primary endpoint of disease control. The second study looked at the use of gemcitabine and erlotinib with and without metformin in patients with locally advanced or metastatic pancreatic cancer. While objective response was the same in both groups, survival at 6 months, overall survival, and progression-free survival were higher in the placebo group than in the treatment group. It is likely that, because of the advanced stage of disease in these patients, the modest benefit if any of metformin was overcome by the burden of disease. This hypothesis is further supported by a recent report of the effect of metformin in a phase II trial of patients with advanced pancreatic cancer. In this open-label, randomized phase II trial, metformin at the dose commonly used in diabetes did not improve outcomes in patients with metastatic pancreatic cancer who were treated with cisplatin, epirubicin, capecitabine, and gemcitabine [32]. Most likely, the impact of metformin use in patients with pancreatic cancer would be best observed in the adjuvant setting after surgical resection or as a chemopreventive agent prior to the development of cancer. Larger studies, ideally multi-institutional looking at the impact of metformin use in resected pancreatic cancer, are indicated. More importantly, prospective studies using metformin in addition to standard chemotherapy in this group of patients would be helpful in determining the role of metformin in the adjuvant setting.
